# Role of Melatonin in the Synchronization of Asexual Forms in the Parasite *Plasmodium falciparum*

**DOI:** 10.3390/biom10091243

**Published:** 2020-08-27

**Authors:** Maneesh Kumar Singh, Bárbara Karina de Menezes Dias, Célia R. S. Garcia

**Affiliations:** 1 Department of Clinical and Toxicological Analysis, Faculty of Pharmaceutical Sciences, University of São Paulo, São Paulo, SP 05508-000, Brazil; maneesh@usp.br; 2 Department of Parasitology, Institute of Biomedical Sciences, University of São Paulo, São Paulo, SP 05508-000, Brazil; bkmdias@gmail.com

**Keywords:** melatonin, Apicomplexa, rhythm, signalling

## Abstract

The indoleamine compound melatonin has been extensively studied in the regulation of the circadian rhythm in nearly all vertebrates. The effects of melatonin have also been studied in Protozoan parasites, especially in the synchronization of the human malaria parasite *Plasmodium falciparum* via a complex downstream signalling pathway. Melatonin activates protein kinase A (PfPKA) and requires the activation of protein kinase 7 (PfPK7), PLC-IP_3_, and a subset of genes from the ubiquitin-proteasome system. In other parasites, such as *Trypanosoma cruzi* and *Toxoplasma gondii*, melatonin increases inflammatory components, thus amplifying the protective response of the host’s immune system and affecting parasite load. The development of melatonin-related indole compounds exhibiting antiparasitic properties clearly suggests this new and effective approach as an alternative treatment. Therefore, it is critical to understand how melatonin confers stimulatory functions in host–parasite biology.

## 1. Introduction

Malaria is a disease associated with a remarkably high mortality rate in its endemic areas, which have subtropical climates. Data collected by the World Health Organization in 2019 using various methods, such as epidemiological, geographic, and demographic, reported an estimated 228 million cases and approximately 4.05 × 10^5^ deaths in more than 80 countries [1]. An estimated 93% of the casualties were associated with sub-Saharan Africa alone, especially those in pregnant women and children below 5 years of age. Although these numbers have consistently fallen over the years, additional and reinvigorated efforts are needed for preventive measures. Most malaria fatalities are associated with cerebral *P. falciparum* infection, but other *Plasmodium* parasites, viz. *P. vivax, P. ovale, P. malariae* and the monkey malaria parasite *P. knowlesi*, can pose opportunistic threats to humans [2].

The life cycle of the human malaria parasite *P. falciparum* spans an insect vector for meiosis and a vertebrate host for mitosis. A female *Anopheles* mosquito injects few sporozoites subcutaneously into the host during blood feeding. These sporozoites primarily establish infection in hepatocytes, remain hidden from the host’s immune system, and divide mitotically into liver-stage merozoites [3]. After their release from the hepatocytes, the merozoites infect red blood cells (RBCs or erythrocytes) to initiate asexual replication. This asexual intraerythrocytic development (IED) occurs when parasites transform through a series of developmental stages, termed ring, trophozoite, and schizont, and at later stages, the progeny merozoites are released to infect more RBCs. Notably, a few parasites transform into sexual gametocyte forms that guarantee the beginning of the sexual cycle after being ingested by the mosquito. The clinical manifestations and pathology of malaria are associated with periodic or recurring fever as a result of blood-stage parasite proliferation, when mature schizonts burst the host’s RBCs. The periodic rupture of the RBCs releases cytosolic debris and parasite metabolites that prompt host responses, causing malarial symptoms. *Plasmodium*-infected patients experience chills and fever, often accompanied by nausea, headache, and fatigue [4]. More importantly, the periodicity of malarial seizures follows a 24 h cycle, i.e., *P. knowlesi* (24 h), *P. vivax* and *P. falciparum* (48 h), and *P. malariae* (72 h) [5]. Four decades ago, researchers found that the IED of the parasites coincided with the host’s body temperature. A study by Hawking et al. shows that the schizogony of *P. knowlesi* or *P. cynomolgi* changed to midnight instead of midday when an infected monkey was subjected to continuous lowering of body temperature for 2–3 days [6]. The same study pointed out that no alteration in cell division was observed in *P. lophure* because it does not exhibit 24 h IED, indicating the rhythmic sensitivity of the host. Similarly, inversion of the nycthemeral cycle of mice changed the schizogony of *P. chabaudi* to midday [7]. It was also shown that the schizogonic cycle of *P. chabaudi* adjusted to the circadian rhythm of the infected mice after 10 days, even after changing the timing of parasite inoculation time [8]. This evidence suggests that the circadian rhythm of the host plays a role in the synchronous IED of parasites. In this review, we focus on the rhythm of parasitism, especially that of the human malaria parasite *P. falciparum.* Over the years, many studies have suggested that the host hormone melatonin is integral in synchronous parasite proliferation. We will also discuss the efforts that have been made to develop indole-derivative compounds showing antagonistic effects on parasite proliferation. Briefly, we will discuss the effect of melatonin in other unicellular protozoan parasites, such as *Toxoplasma gondii* (toxoplasmosis), *Leishmania* (leishmaniases), and *Trypanosomes* species (Chagas’ and sleeping sickness).

## 2. Melatonin-Dependent Rhythm in Parasites

Melatonin (*N-acetyl-5-methoxytryptamine*) is an indoleamine compound that was first extracted from the bovine pineal gland and has also been reported in the peripheral nerves of other mammals, including humans [9]. Later, endogenous production of melatonin was also reported in plants, bacteria, unicellular organisms, and invertebrates [10,11,12]. It was speculated that melatonin was primarily present in primitive bacteria (cyanobacteria and α-proteobacteria); over the course of evolution, these bacteria cohabited endosymbiotically in early prokaryotes and later became universally distributed in other organisms (reviewed in [13]). The melatonin level in the blood is controlled by day/night exposure; it is high at night when it is secreted from the pineal gland, and its level drops during the day. In animals, melatonin biosynthesis begins after tryptophan, a necessary precursor present in the bloodstream, enters the pineal gland. Once tryptophan reaches the pineal gland, it is hydroxylated to form 5-hydroxytryptophan (5-HTP) and then decarboxylated to produce serotonin. Arylalkylamine *N-acetyltransferase* (AANAT), which is active during the night, acetylates serotonin and converts it to *N-acetylserotonin* (NAS). Then, the enzyme hydroxyindole-o-methyltransferase (HIOMT) methylates the hydroxyl group of NAS to produce melatonin. The whole melatonin synthesis process is regulated by β-adrenergic receptor (β-AR) activation by norepinephrine (NE), which activates protein kinase A (PKA) via cAMP elevation. The suprachiasmatic nucleus (SCN) perceives light/dark information to control the amount of NE in the day via proteasomal degradation [14]. The primary function of melatonin was thought to be nullifying the adverse effects of free radicals and reducing receptor-independent oxidative stress, possibly for two potential reasons. First, it can easily cross cellular membranes due to its hydrophobic nature; second, it can interact with various ROS and donate an electron or a hydrogen atom [15]. Melatonin is known to regulate the circadian rhythm through the measurement of the day/night interval [16]. Its metabolites have been found to control various physiological processes, such as sleep, vasomotor regulation, anti-excitatory activities, immunomodulation and antioxidant properties [17]. The role of the circadian rhythm has been extensively studied in various organisms, including bacteria, fungi, plants, and animals [18,19,20]. It has been estimated that approximately 10% of the genomes of higher vertebrates, such as mammals, are under circadian control [21].

As in higher animals, it has been found that circadian rhythm plays crucial roles in several parasites to support transmission, an important phenomenon that enables the parasites to acquire a new host. This process is more important for *Plasmodium* parasites, considering their short time window from erythrocyte rupture to invasion, because after RBC rupture, the parasites are exposed to the host defence system. To avoid prolonged exposure to the host immune system, parasite egress is very synchronous; to accomplish this, *Plasmodium* spp. follow cycles that are multiples of 24 h [22]. The proliferation of asexual malaria parasites and their host-transmission efficiency are severely affected when the rhythm of the host and parasite during IED is perturbed; however, parasites quickly adapt to changes in the new environment [23,24]. Despite the periodic febrile waves generated in *Plasmodium*-infected hosts, the driving cues related to this periodicity are largely unknown, and if there is a host cue, then how it regulates periodicity during IED is unknown. Interestingly, it is known that *Plasmodium* parasites match the host’s rhythm after establishing an infection but become asynchronous when grown in vitro, indicating a host-derived signal. The role of the circadian rhythm was overlooked until Hotta et al. for the first time studied the role of melatonin in the synchronization of IED, both in vitro and in vivo, in *Plasmodium* [25]. In this study, when asynchronous *P. falciparum* parasites were grown in vitro with 100 nM melatonin, the parasite cycle was accelerated towards a more mature schizont stage. Similarly, murine *P. chabaudi* parasites lost synchrony in mice with surgically removed pineal glands or mice injected with the melatonin receptor antagonist luzindole, and synchrony was restored by administering melatonin [25]. Interestingly, two murine parasites, *P. berghei* and *P. yoelii*, do not display melatonin-dependent synchronization in vivo [26]. The difference in the parasites showing melatonin response is the ability of melatonin to mobilize cytosolic Ca^2+^ ([Ca^2+^]_cyt_). We will discuss the molecular mechanism of melatonin-induced signalling in the next section. Additionally, melatonin modulates the expression of genes related to the ubiquitin/proteasome system (UPS) in both mammals and *P. falciparum* [27,28,29]. It is evident that the UPS is fundamental in regulating the cell cycle and transcriptional activity of cells [30,31]. Not only melatonin, but also its precursors 5-hydroxytryptophan, serotonin and *N-acetylserotonin* modulate IED in *P. falciparum*, increase mature schizont percentage and mobilize [Ca^2+^]_cyt_ [32] (Table 1). Apart from that, the melatonin catabolic product *N1-acetyl-N2-formyl-5-methoxykynuramine* (AFMK) accelerates *P. falciparum* and *P. chabaudi* synchronization via [Ca^2+^]_cyt_ mobilization [33]). This leads to a new question of whether other compounds having an indole ring are also capable of affecting IED in these parasites. To this end, the indole-derivative plant hormone indole-3-acetic acid (IAA) was studied, and it was found that IAA modulates neither the IED of the parasites nor UPS gene expression [34]. In addition, few synthetic indole derivatives can regulate the cell cycles of parasites [35], suggesting that not all indole derivatives confer cell cycle modulation. More recently, RNA-seq analysis of melatonin-treated *P. falciparum* parasites revealed 38 differentially expressed genes, among which several genes were related to post-translational modification (PTM), Zn-binding proteins and nucleic acid-binding proteins [36].

Another important aspect of melatonin is its synthesis in the mitochondria [37], cellular powerhouses that produce ATP, generate free radicals and induce apoptosis. The production of melatonin in the mitochondria provides a buffer against oxidative stress to prevent the mitochondria from collapsing. Additionally, evidence supports the presence of the melatonin receptors MT1 and MT2 in the mitochondria, which may provide protection against ischaemia-induced brain injury [38,39]. Mitochondria also act as Ca^2+^ reservoirs, along with the ER. It has been shown that mitochondria take up Ca^2+^ very efficiently due to their proximity to the ER, so they can easily sense the Ca^2+^ microdomain as a result of IP_3_ hydrolysis [40]. Although the role of melatonin in Ca^2+^ homeostasis in relation to mitochondria has mostly been explored in the mammalian system, how melatonin affects parasite mitochondria is still unknown. The genome sequence of *Plasmodium* has not revealed any melatonin receptor, making it difficult to investigate melatonin signalling in relation to Ca^2+^ homeostasis in mitochondria. However, our group has shown that mitochondria participate in Ca^2+^ homeostasis through the reversible uptake of ER-released [Ca^2+^]_cyt_ [41]. It would be interesting to investigate how melatonin modulates mitochondrial Ca^2+^ and whether it has beneficial effects in parasites.

## 3. Melatonin Triggers a Signalling Cascade in Plasmodium Parasites

**Figure 1:**
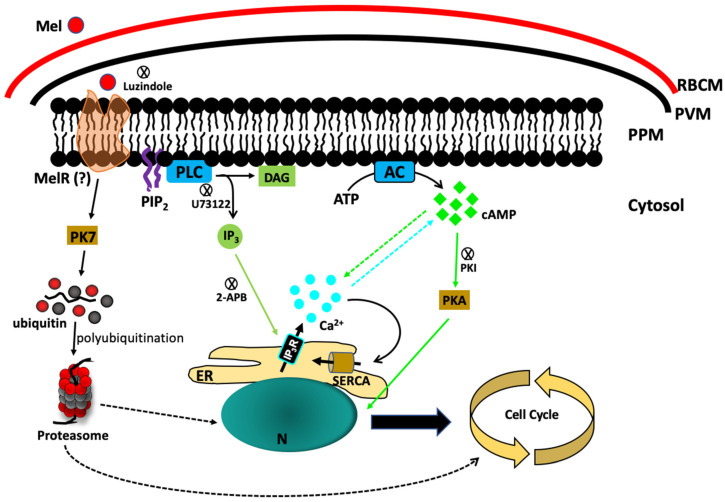
Melatonin signalling in *P. Falciparum*. Melatonin stimulates the cleavage of PIP_2_ by phospholipase C (PLC) to produce IP_3_, which activates IP_3_R to release Ca^2+^ in the cytosol. Simultaneously, melatonin also activates the production of cAMP, which triggers the downstream PfPKA signalling cascade. On the other hand, the orphan kinase PfPK7 is upregulated by melatonin and is linked with the parasite’s proteasomal activation. RBCM—RBC membrane; PVM—Parasitophorous vacuole membrane; PPM—Parasite plasma membrane; Mel—Melatonin; MelR—Melatonin receptor (hypothetical); AC—Adenylyl cyclase; PLC—Phospholipase C; PIP_2_—Phosphatidylinositol-4,5-biphosphate; IP_3_—Inositol-1,4,5-triphosphate; DAG—Diacylglycerol; ER—Endoplasmic reticulum; N—Nucleus; SERCA—sarco/endoplasmic reticulum Ca^2+^—ATPase.

The molecular mechanism by which melatonin controls the signalling cascade to regulate the parasite cell cycle is still not fully understood. When Hotta et al. showed that melatonin accelerates the IED, they also noticed that melatonin triggered the release of Ca^2+^ from the internal stores of *Plasmodium* and activated the PLC-IP_3_ signalling pathway. The generation of IP_3_ was triggered by the parasite’s phospholipase C (PLC) enzyme, and blocking PfPLC with U73122 obliterated the melatonin-induced Ca^2+^ increase [25] (Figure 1). To further strengthen the melatonin hypothesis, it has been shown that parasites that are not sensitive to melatonin-induced synchronization are not able to elicit [Ca^2+^]_cyt_ release from the internal stores [26]. In another study, Vaid et al. showed that the PLC blocking compound U73122 inhibited Ca^2+^ release and blocked PfPKB activation, suggesting that Ca^2+^ acts upstream of the PfPKB signalling cascade in *P. falciparum* [48]. The dispensable role of phosphoinositide-specific PLC in Ca^2+^ generation was further suggested by Raabe et al., who performed a transcriptional analysis of PLC in the blood stage of *P. falciparum*. They found steady PfPLC expression in the blood stage and showed a transient increase in expression in late schizonts [49]. The first direct study by Alves et al. reported a relationship between melatonin and the PLC-IP_3_ pathway, where melatonin triggers IP_3_ production by activating PfPLC and opens ER-localized IP_3_-sensitive Ca^2+^ channels [50]. Alves et al. used cell-permeant caged IP_3_ in intact *P. falciparum*-infected RBCs that releases IP_3_ upon UV light exposure. They found that UV light exposure of the parasites caused photolysis of caged IP_3_ and elicited a rapid and transient increase in [Ca^2+^]_cyt_, but melatonin stimulation before the flash photolysis of the caged IP_3_ abolished the [Ca^2+^]_cyt_ increase [50]. It was also reported that both *P. falciparum* and *P. chabaudi* sustain intracellular Ca^2+^ stores, and isolated and permeabilized *P. chabaudi* showed IP_3_-dependent Ca^2+^ discharge [51]. It is interesting to consider that either a putative melatonin receptor or a putative IP_3_-receptor (IP_3_R) has remained unidentified in *P. falciparum*. However, IP_3_R has been characterized in two different protozoan parasites, *T. cruzi* [52] and the food vacuoles of *T. brucei* [53]. These studies independently showed that either disrupting or inhibiting the IP_3_ receptor resulted in impaired growth and infection rates.

At the molecular level, melatonin has been shown to establish cross-talk between cAMP and Ca^2+^. A membrane-permeant 3′,5′-cyclic monophosphate N6-benzoyl/protein kinase A activator (6BZ-cAMP) exhibited dual specificity, activated protein kinase A (PfPKA) and increased [Ca^2+^]_cyt_ levels. Melatonin-induced cAMP was blocked by the PLC inhibitor U73122 or by blocking PfPKA with a peptide inhibitor of PKA (PKI) that prevents increases in [Ca^2+^]_cyt_, indicating that PKA is required for Ca^2+^ induction [45] (Figure 1). To elucidate how melatonin signalling occurs in parasites, Beraldo et al. found that the IP_3_R antagonist 2-aminoethoxydiphenyl borate (2-APB) nullifies the effects of melatonin on the cell cycle and [Ca^2+^]_cyt_. They have also reported that Ca^2+^ influx is linked to sustained Ca^2+^ mobilization from intracellular organelles upon treatment with the cell-permeant cAMP analogue 8BrcAMP, which is referred to as capacitative Ca^2+^ entry (CCE) [54]. This store-operated Ca^2+^ entry (SOCE) was also blocked by two 2-APB derivatives, DPB162-AE and DPB163-AE, but this occurred at high concentrations in the presence of melatonin, suggesting a differential activation mechanism of SOCE in parasites [55]. Pecenin et al. have also shown that *P. falciparum* failed to survive in vitro when transfected with IP_3_-sponge or IP_3_ binding domain (IRIS)-containing plasmids [55]. These findings provide additional evidence that melatonin-induced signalling is associated with the IP_3_/Ca^2+^ pathway and critical for parasite IED.

Another piece of evidence came to light during a study of the orphan kinase PfPK7, which has C-terminal homology with MEK and N-terminal homology with fungal PKA [56]. The PfPK7 knockout (PfPK7^−^) parasites were not able to exhibit melatonin-induced synchronization and [Ca^2+^]_cyt_ rise, but complementation with a functional copy of PfPK7 reverted the parasites towards melatonin sensitivity. Additionally, the upregulation of 14 UPS genes, including E1, E2, E3, ubiquitin-like, deubiquitinase, and proteasome subunits, by melatonin has been shown in wild-type *P. falciparum* and abolished in PfPK7^−^ parasites [28]. Further evidence that PfPK7 is pivotal for melatonin signalling came from two studies that performed RNA-seq analysis and examined mitochondrial genes related to mitochondrial fission [36,47]. Lima et al., in the RNA-seq study, compared the effects of 5 h melatonin treatment on differentially expressed genes in wild-type *P. falciparum* 3D7 and in PfPK7^−^ parasites. They found that 5 h melatonin treatment at the trophozoite stage resulted in 38 differentially expressed genes in wild-type parasites but not in PfPK7^−^ or untreated parasites. Additionally, 6 h cAMP treatment differentially modulated 75 genes in rings, 101 genes in trophozoites, and 141 genes in schizonts [36]. A study by Scarpelli et al., however, showed the relevance of PfPK7 to the alteration of mitochondrial fission genes PfFIS1, PfDYN1, and PfDYN2 upon melatonin treatment. The relative expression of these genes was significantly altered in the presence of melatonin in wild-type parasites, but the effect was abolished in PfPK7^−^ parasites [47]. Increased transcription of UPS genes, especially ubiquitin-activating enzyme E1 and ubiquitin ligase E3, along with the expression of the transcription factor PfNF-YB, was observed in *P. falciparum* parasites in the presence of melatonin [57]. This evidence suggests the pivotal role of melatonin in the signalling cascade that controls the gene expression leading to parasite maturation. Considering all the above mentioned factors, it became important to investigate the physiological and molecular aspects of Ca^2+^ signalling in these parasites, including how these parasites sense external cues and activate signalling cascades mediated by a transient increase in secondary messengers, viz. cAMP and/or [Ca^2+^]_cyt_, from their internal stores. It would be interesting to investigate genes that modulate *P. falciparum* growth and are related to circadian rhythm.

In mammalian systems, melatonin acts through specific receptors to confer downstream effects in cells. Receptors for melatonin (MT1 and MT2) have been identified in mammalian systems as G-protein coupled receptors (GPCRs) [58] that are associated with downstream signalling events to affect the levels of cytosolic secondary messengers, generally decreasing cAMP or cGMP and increasing IP_3_ or Ca^2+^ [59]. To identify melatonin receptors in the *Plasmodium* genome, a genome-wide search was performed. Madeira et al. identified four GPCR-like or serpentine receptor-like (SR) genes, PfSR1, PfSR10, PfSR12, and PfSR25, in *P. falciparum*, which are expressed mostly during the IED of this parasite [60]. The functional characterization of these GPCR-like proteins is still under investigation. Among these GPCR-like proteins, PfSR25 has been characterized as an external K^+^ sensor during parasite egress that triggers the release of [Ca^2+^]_cyt_ and enables the parasite to adapt under stress conditions [61]. It is not clear how PfSR25 triggers the signalling cascade, since GPCRs are coupled to trimeric G-proteins that are absent in the *Plasmodium* genome. Interestingly, an RBC protein, Gα_s_, along with β-adrenergic receptor (β-AR), is recruited on the vacuolar membrane of the parasite. Stimulation of β-AR activates Gα_s_ by cAMP production and increases the infection percentage, while competitive inhibition of Gα_s_ with peptides reduces *P. berghei* parasitemia [62]. This study indicates that parasites require GPCR-mediated signalling, and this area is central for the development of new therapies against malaria. Another *Plasmodium* GPCR-like protein, PfSR10, exhibits a circadian transcription profile with peak expression at two time points during the IED, the first after 8 h and the second after 32 h post-invasion. Disrupting its orthologous gene in *P. chabaudi*, PcSR10, shortens the IED by 2–3 h in mice [63]. Similarly, it has been reported that *Plasmodium* parasites exhibit intrinsic rhythm in more than 75% genes during IED, increasing the complexity of host–parasite periodicity [64,65].

## 4. Melatonin Confers Protective Immunity against Parasitic Infection

Early pathogenic infection must be recognized rapidly by the host innate immune system to clear invasive microbes. The innate immune response provides the first line of defence against pathogens and is based on pattern recognition receptors (PRRs) that recognize pathogen-associated molecular patterns (PAMPs). PRRs are classified into membrane-associated toll-like receptors (TLRs), C-type lectin receptors (CLRs), cytoplasmic non-membranous NOD-like receptors (NLRs) and RIG-I-like receptors (RLRs) (reviewed in [66]). PAMPs consist of a different range of molecules, including lipids, carbohydrates, nucleic acids or their combinations. Once PAMPs are recognized by PRRs, innate immune cells activate specific PAMP-related signalling cascades via cytokine and chemokine production. More importantly, these features are unique to pathogens and essential for survival. However, the majority of the information available is restricted to viral and bacterial infection, but a few studies have revealed how PAMPs work during parasitic infections. However, recent advancements in this area have revealed the PAMPs associated with Apicomplexan and Trypanosomatid protozoan parasites. In the case of *Plasmodium* parasites, glycosylphosphatidylinositol (GPI) expressed on the merozoite surface is recognized by the TLR1-TLR2 heterodimer [67,68] and activates MAPK and NF-κB pathways [69]. Similarly, growing malaria parasites digest haemoglobin to produce inert haemozoin moieties, which are recognized by TLR9 by an unusual process where haemozoin forms a complex with the parasite DNA and is presented to TLR9 [70]. Plasmodium-derived haemozoin and DNA also activate cytosolic NLRP3 through Lyn and Syk kinases and induce the production of the cytokines IL-1 and IL-18 [71,72]. In *T. gondii*, GPI anchors and profilin-like proteins (PFTGs) are recognized by TLR2, TLR4 and TLR11, respectively [73,74]. TLR11 recognition of PFTG induces IL-12 production that is dependent on myeloid differentiation factor 88 (MyD88) [74]. Despite this evidence, we have very elementary knowledge of whether the hormone melatonin plays any protective role in the host during parasitic infection and enhancement of innate immunity. Therefore, we directed our focus towards studies that reveal how melatonin induces or enhances the host response during parasitic infection.

Similar to *Plasmodium*, other protozoan parasites, such as *Trypanosoma* spp., *T. gondii*, and *Leishmania* spp. respond to the host hormone melatonin. It is apparent that melatonin modulates the immune system via receptor recognition. Melatonin receptors have been identified on CD4^+^ and CD8^+^ cells and B lymphocytes [75]. CD4^+^ cells induce delayed-type hypersensitivity (DTH) that activates proinflammatory cytokine production, while CD8^+^ cells exert a cytotoxic effect; both protect against intracellular protozoa [reviewed in [76]. In mice infected with *T. gondii*, melatonin prompts the cellular immune response by galvanizing CD4^+^ and CD8^+^ lymphocyte production, causing a surge in proinflammatory cytokines [77]. *T. gondii*-infected mice show increased nitric oxide (NO) levels in the plasma, which have both immunoprotective and immunomodulatory roles in chronic infection. However, higher NO production has a neurotoxic effect and promotes central nervous system (CNS) degeneration in infected mice [77]. Melatonin can counteract the NO level by reducing induced NOS (iNOS) activity to provide beneficial support during toxoplasmosis. In this regard, it was shown that NO levels increased after *T. gondii* infection, especially in pinealectomized rats [78]. Subsequently, it was found that cellular infiltration of lymphocytes and CD cells increased in melatonin- and zinc-supplemented rats, and an adjunctive therapy to treat *Toxoplasma* retinochoroiditis in immunocompromised patients was proposed [79]. In a recently published report on a monkey kidney epithelial cell line, LLC-MK2 showed protection against *T. gondii* with melatonin treatment. Despite a lack of alteration in cell viability, Machado et al. found that melatonin treatment altered the invasive tachyzoite shape, which was associated with ruptured plasma membrane and cytoplasmic leakage [80].

Similarly, another vector-borne parasite, *Trypanosoma cruzi*, has serious effects if left untreated. It was found that proinflammatory cytokines play protective roles against *T. cruzi* infection and activate macrophages during acute infection. Activated macrophages trigger two responses: first, they increase NO production to protect against early infection, and second, they enhance the cellular response by activating Th1 cells [81,82]. These responses involve the production of various proinflammatory cytokines, such as Interferon-γ (IFN-γ), tumour necrosis factor-α (TNF-α), and interleukins (IL-2, IL-12), that help in parasite clearance. The role of melatonin in *T. cruzi* infection, in which it stimulates the host immune response, has been studied. Concomitant treatment with 5 mg/kg body weight melatonin in rats infected with *T. cruzi* enhances the rat immune response and elevates IFN-γ, TNF-α, IL-2, and IL-12. along with increasing peritoneal macrophage numbers [83,84]. In infected rats, melatonin administration also reduced parasite burden, tissue destruction and inflammatory cells [84]. Increased cytokine levels and reduced parasitaemia were also detected in infected rats when melatonin was administered along with the anti-inflammatory drug meloxicam [85] and dehydroepiandrosterone (DHEA), a secretory substance from the adrenal cortex [86]. Oxidative stress-induced pathophysiology during *T. cruzi* infection was observed through lipid peroxidation (LPO) and in the myocardium of infected animals [87,88]. Melatonin treatment of infected rats protects against parasites by counteracting NO and TNF-α levels [89] and enhances the immune response by increasing the production of the intracellular cytokines IFN-γ, TNF-α, IL-4, IL-10 and IL-17 [87,90]. Another *Trypanosoma* parasite, *T. brucei*, causes sleeping sickness and exhibits an intrinsic circadian clock, and approximately 10% of its genes are in periodic rhythm during in vitro growth [91]. However, alteration in the melatonin secretion rhythm and its binding to melatonin receptors have been observed in *T. brucei*-infected mice, suggesting the clinical pathology associated with the disease, where the sleep cycle of the host is affected [92].

Consistent with the above-described phenomena, melatonin could also regulate the infectivity of leishmaniases caused by protozoan parasites of *Leishmania* spp. It was shown that the melatonin rhythm was unaffected in *L. amazonensis*-infected hamsters, and melatonin provided protection against parasites injected in the night. Similarly, administering melatonin during light reduces parasite burden and lesion progression. In another study, the authors showed that melatonin reduces arginine uptake and polyamine availability, which are essential for parasite replication [93]. *Leishmania* parasites proliferate inside phagocytic cells such as macrophages and use polyamines for their growth [94]. Arginine uptake in macrophages is facilitated by a cationic amino acid transporter (CAT-2B) and then converted to either polyamine by arginase I or NO by NOS2, where NO is cytotoxic for *Leishmania*. Interestingly, this study showed that *Leishmania* parasites can upregulate CAT-2B expression to increase arginine uptake by macrophages, which can play either protective or proliferative roles [95]. Moreover, melatonin has recently been shown to modify the transcriptomic profile of miRNAs in *Leishmania*-infected macrophages. In this study, Fernandes et al. showed that melatonin impaired *L. amazonensis* infection by altering miRNA expression, which in turn modulated *Nos2*, *Tnf*, *Mcp-1/Ccl2*, and *Rantes/Ccl5* expression, correlating with the activation of macrophages and cell recruitment to the inflammation site in infected mice [96].

All the above pieces of evidence suggest that melatonin provides positive feedback during parasitic infection and protects the host against severe parasitic load by increasing proinflammatory cytokines and counteracting oxidative stress.

## 5. Indole-Derivative Compounds as Antimalarials

Very limited numbers of drugs are available to treat malaria, and the emerging drug-resistant strains of *P. falciparum* in Southeast Asia are rapidly compromising existing malaria prevention strategies [97]. Based on their sites of action, most antimalarial drugs are divided into six classes, viz. 4-aminoquinolines (amodiaquine, chloroquine), 8-aminoquinolines (primaquine), artemisinin, antifolates, arylamino alcohols, and respiratory chain inhibitors [98]. Chloroquine (CQ) is the most widely used gold standard drug for treating uncomplicated malaria, but its prolonged use has resulted in the emergence of resistant parasites, making it less effective [99,100,101]. Understanding the biology of the parasite is essential to develop new drugs to counteract parasite growth, since the current therapy is rapidly failing due to the emergence of resistant parasite strains [102,103]. We already know that melatonin has strong anti-inflammatory, antioxidative and immunoregulatory properties and thus maintains the balance between innate and adaptive immunity. Alongside CQ, melatonin is gaining strong support to mitigate mild symptoms of the current global COVID-19 pandemic caused by the novel coronavirus SARS-CoV-2 [104]. It is very interesting that CQ, hydroxychloroquine (HCQ) and melatonin share very similar chemical structures and a common interaction site for human quinone reductase 2 (hQR2, or NQO2), also known as melatonin receptor 3 (MT3) (reviewed in [105]). Evidence suggests an interaction between CQ and melatonin in vitro, where CQ blocked melatonin-induced autophagy [106]. However, no such data are available in the malarial model to implicate the direct role of melatonin and CQ in either synergistic or antagonistic effects. It would be interesting to study the kinetic behaviour of these molecules to further investigate their application in malaria treatment. Indole compounds have shown antimalarial activity in vitro, representing a potential new class of antimalarials and raising the interest of multiple groups to study the effects of this class of compounds and to elucidate the mechanism by which these indoles impair the development of parasites.

Agarwal et al. synthesized a series of 24 substituted indole derivatives and tested the antimalarial activity of each compound. These synthetic compounds presented an indole fraction and a pyrimidine fraction with cyclic amine substituents in the pyrimidine ring. The results indicated that substitution at the 2-position of the pyrimidine ring is important for antimalarial activity and that the substituent *N-methyl* piperazine presented better efficiency than piperidine, morpholine and pyrrolidine. Six compounds showed an MIC_50_ of 1 µg/mL [107]. Chierrito et al. investigated the antimalarial potential of *Aspidosperma olivaceum*, a plant used to treat human diseases in the tropics, focusing on monoterpene indole alkaloids present in the plant. The authors tested extracts from the bark and leaf and monoterpene indole alkaloids isolated from stem bark and leaf of *A. olivaceum* (aspidocarpine, uleine, apparacine and *N-methyl-tetrahydrolivacine*) against the chloroquine-resistant *Plasmodium falciparum* strain W2 in vitro. The results obtained showed that aspidocarpine was the most promising compound, presenting IC_50_ values of 5.4 µµg/mL by the [3 H]-hypoxanthine method and 4.4 µg/mL by HRPII assays. This compound also presented the highest selection index (SI) of 56 and presented low toxicity against HepG2 cells. In addition, the authors also showed that an acidic extract from *A. olivaceum* leaves reduced parasitaemia in mice infected with *P. berghei* [108]. In another study, of an *Aspidosperma* plant by Dolabela et al., extracts from the trunk bark of *Aspidosperma parvifolium* were tested against chloroquine-sensitive (3D7) and chloroquine-resistant (W2) strains of *P. falciparum* in vitro. Uleine, a monoterpene indole alkaloid obtained from an ethanol extract of *A. parvifolium* trunk bark, was the most promising compound tested, with IC_50_ values of 0.75 µg/mL in *P. falciparum* W2 and 11.90 µg/mL in 3D7 obtained by the microscopic method, 8.78 µg/mL obtained by the [3 H]-hypoxanthine method, and 2.95 µg/mL obtained by HPRII assay in W2 parasites [109].

Shuck et al. investigated the ability of a series of ten indole compounds analogous to melatonin to impair the synchronization of the *P. falciparum* erythrocytic cycle triggered by melatonin and parasite growth. The authors showed that eight out of ten compounds were able to impair the melatonin effect on synchronicity at a concentration of 500 nM of each compound combined with 100 nM of melatonin. Furthermore, the authors identified three compounds with antimalarial activity against *P. falciparum* in vitro [35]. The compound with the highest activity (Compound 14) showed an IC_50_ of 2.93 µM. In another study, Luthra et al. tested five indole-based C_2_-arylimino tryptamine derivative compounds in the melatonin pathway. The authors identified compound 2a as the most potent, since it inhibited 47% of *P. falciparum* growth at 5 µM. Fourteen new compounds were then synthesized based on the lead compound of the study (2b). Five compounds (2g, 2i, 2j, 2k and 2p) presented antimalarial activity against *P. falciparum* in vitro and presented IC_50_ values of 4.28 µM, 0.89 µM, 0.74 µM, 2.73 µM and 0.73 µM, respectively. In addition, these compounds also showed efficacy against the chloroquine-resistant strain RKL9 [110]. To elucidate whether these compounds act in the pathway triggered by melatonin in *P. falciparum*, they were tested against an asynchronous culture of parasites, and compound 2j was able to impede the effect of the hormone and bind strongly to the mammalian melatonin receptor MT1 [110]. In a study of structure and activity, Lunga and colleagues synthesized a series of indole compounds with substituents in C5, C2, and C3. The authors pointed out that methylation at C2 decreased the activity of the compounds. For the C5 substitutions, hydrophobicity was more important than electron-withdrawing capacity, and substitutions with chloride, fluorine, methyl, methoxy, or nitrile moieties pointed to the existence of an optimal substituent size, with the best result obtained with a chloride radical in C5 [111]. The most promising compounds were further tested against *P. falciparum* NF54 and strain K1, which are resistant to chloroquine. Compound 14 was the most active compound against the NF54 and K1 strains of the parasite. Pasaje et al. identified two tryptophanyl-tRNA synthetases in *P. falciparum*, one in the apicoplast (TrpRS^Api^) and the other in the cytoplasm (TrpRS^Cyt^). Tryptophanyl-tRNA synthetase is essential for protein translation, since it combines tRNA with tryptophan. Interestingly, the authors tested the natural indole compound indolmycin, a tryptophan analogue, and two indolmycin analogues against *P. falciparum* in vitro. The results show that indolmycin elicits delayed death in *P. falciparum*, inhibiting parasite growth through the second cell cycle after treatment with an IC_50_ of 1.7 µM. The results obtained indicated that indolmycin acts against the parasite by targeting TrpRS^Api^ and impairing apicoplast function [112]. In another study, Dangi et al. tested a library of indole compounds based on the natural products usambarine and aspidocarpine. The authors identified two compounds (6 and 7) able to inhibit parasite growth at 80% with 50 µM and obtained IC_50_ values of 32.4 µM and 21.8 µM for compounds 6 and 7, respectively. Furthermore, the results showed that the parasite life cycle was arrested at the trophozoite stage by compound 7. In addition, the authors showed that compound 7 affects the Na^+^ balance in the parasite and that compounds 6 and 7 fit into the binding pocket from PfATP4, a P-type cation translocation ATPase, and interacts with amino acid residues in the active site of the enzyme in silico [113].

## 6. Concluding Remarks and Future Perspectives

Vector-borne parasitic diseases still pose a major threat to developing nations despite global research efforts to increase preventive measures. Malaria has emerged as a major problem causing severe setbacks to the global economy. Studies suggest that coinciding with host rhythm is advantageous for parasite proliferation in the host. Periodicity ensures a greater survival efficiency and growth rate of the parasites, especially by allowing them to avoid the host immune response. Recent studies show that malaria parasites have an intrinsic periodic rhythm for more than 80% of the genes expressed during IED [64,65] but do not deny the possibility of host cues. Melatonin, an ancient indolamine compound, has very complex physiological properties in all organisms and has also been implicated in host–parasite interactions. It plays a fundamental role in controlling parasite replication and host survival. However, malaria parasites adapt well to the host circadian rhythm, and melatonin modulates the synchrony of parasites both in vivo and in vitro. This phenomenon implicates the evolutionary adaptability of the parasite, allowing it to escape from host immune surveillance given that free merozoites have a low survival rate if they do not invade immediately; hence, synchronous rupture gives them a better opportunity to invade circulating RBCs. Melatonin-related analogues show antagonistic properties against *Plasmodium* parasites, suggesting that they may have great importance as a novel therapeutic approach against these malaria parasites, which affect millions of people worldwide.

## Figures and Tables

**Table 1:** Effects of indole-related compounds on *Plasmodium* parasites.

Compounds	Action
Tryptophan	*P. falciparum*-infected erythrocytes exhibit higher tryptophan uptake [42]. Inhibition of tryptophan catabolism during *P. yoelii* infection partially protects mice against lethal infection [43]
5-Hydroxytryptophan	Activates PCL/IP_3_ pathway to increase cytosolic Ca^2+^ and promotes cell cycle acceleration to increase schizont percentage in vitro in *P. falciparum* [32]
Serotonin	Activates PCL/IP_3_ pathway to increase cytosolic Ca^2+^ and promotes cell cycle acceleration to increase schizont percentage in vitro in *P. falciparum* [32]
*N-Acetylserotonin*	Activates PCL/IP_3_ pathway to increase cytosolic Ca^2+^ and promotes cell cycle acceleration to increase schizont percentage in vitro in *P. falciparum* [32,44]
Melatonin	Activates PCL/IP_3_ pathway to increase cytosolic Ca^2+^ and promotes cell cycle acceleration to increase the schizont percentage in vitro in *P. falciparum* [25,32,44]. Modulates neither parasite maturation nor cytosolic Ca^2+^ in *P. yoelii* and *P. berghei* [26]. Induces cAMP-dependent kinase PfPKA [45]. Upregulates UPS genes [46]. Promotes differential gene expression in *P. falciparum* trophozoite stage parasites [36]. Activates mitochondrial genes PfFIS1, PfDYN1, and PfDYN2 [47].
Indole-3-acetic acid (IAA)	Modulates neither the IED of the parasites nor UPS gene expression [34].
